# Characteristics and treatment outcome in a prospective cohort of 639 advanced high-grade digestive neuroendocrine neoplasms (NET G3 and NEC). The NORDIC NEC 2 study

**DOI:** 10.1038/s41416-025-03054-w

**Published:** 2025-05-17

**Authors:** Halfdan Sorbye, Geir Olav Hjortland, Lene Weber Vestermark, Morten Ladekarl, Johanna Svensson, Anna Sundlöv, Eva Tiensuu Janson, Herish Garresori, Eva Hofsli, Christian Kersten, Hege Elvebakken, Per Pfeiffer, Siren Morken, Jorg Assmus, Inger Marie Bowitz Lothe, Elizaveta Tabaksblat, Ulrich Knigge, Anne Couvelard, Aurel Perren, Seppo W. Langer

**Affiliations:** 1 Cancer Clinic, Haukeland University Hospital, Bergen, Norway; 2 Department of Clinical Science, University of Bergen, Bergen, Norway; 3 Department of Oncology, Oslo University Hospital, Oslo, Norway; 4 Department of Oncology, University Hospital of Southern Denmark, Esbjerg, Denmark; 5 Department of Oncology, Aarhus University Hospital, Aarhus, Denmark; 6 Department of Oncology, Clinical Cancer Research Center, Aalborg University Hospital, and Department of Clinical Medicine, Aalborg University, Aalborg, Denmark; 7 Department of Oncology, Sahlgrenska University Hospital, Gothenburg, Sweden; 8 Department of Oncology, Skåne University Hospital, Lund, Sweden; 9 Department of Medical Sciences, Section of Endocrine Oncology, Uppsala University, Uppsala, Sweden; 10 Department of Oncology, Stavanger University Hospital, Stavanger, Norway; 11 Department of Oncology, St.Olavs Hospital, Trondheim, Norway; 12 Department of Research, Hospital of Southern Norway, Kristiansand, Norway; 13 Department of Oncology, Ålesund Hospital, Møre og Romsdal Hospital Trust, Ålesund, Norway; 14 Department of Oncology, Odense University Hospital, Odense, Denmark; 15 Centre for Clinical Research, Haukeland University Hospital, Bergen, Norway; 16 Department of Pathology, Oslo University Hospital, Oslo, Norway; 17 Department of Surgery C and Endocrinology PE, Rigshospitalet, Faculty of Health Science, University of Copenhagen, Copenhagen, Denmark; 18 Department of Pathology, Bichat Hospital, Paris, France; 19 Institute of Tissue Medicine and Pathology, University of Bern, Bern, Switzerland; 20 Department of Oncology, Rigshospitalet, Copenhagen, Denmark; 21 Department of Clinical Medicine, University of Copenhagen, Copenhagen, Denmark

**Keywords:** Outcomes research, Neuroendocrine cancer

## Abstract

**Background:**

Digestive high-grade neuroendocrine neoplasms (HG-NEN) are rare and classified as neuroendocrine carcinomas (NEC) or neuroendocrine tumours G3 (NET G3), and differ in clinical and molecular characteristics, response to treatment and prognosis.

**Methods:**

Prospective multicenter study registering clinical data on patients with digestive HG-NEN. Treatment outcome in patients with advanced disease was compared after centralized pathological re-evaluation.

**Results:**

427 NEC and 117 NET G3 received palliative chemotherapy. Immediate progression rate was 41% and 24%, progression-free survival (PFS) 3.4 m and 7.4 m, overall survival (OS) 7.4 m and 21.8 m for NEC and NET G3, respectively. Significant factors for OS in NEC were performance status (PS), Ki-67 > 55%, alkaline phosphatase (ALP), age, sex and for PFS colorectal primary and PS. NEC Ki-67 < 55% had similar OS comparing treatment. Significant factors for OS in NET G3 were platinum-based treatment, PS, age and ALP, and for PFS platinum-based treatment.

**Conclusions:**

Survival was shorter than expected in this unique population-based cohort of advanced digestive HG-NEN, likely due to inclusion of elderly and patients with poor PS. Several novel prognostic factors were identified for NEC and NET G3. An initial sub-effective platinum-based treatment for NET G3 could not be compensated by later-line treatment.

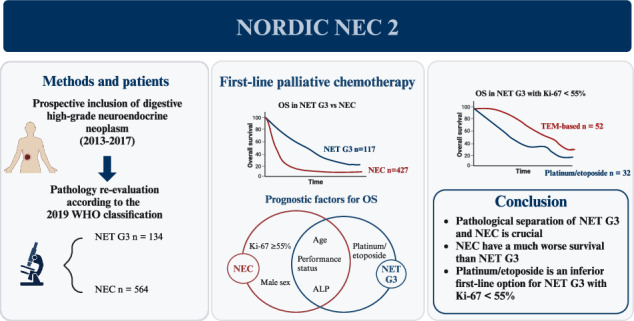

## Introduction

Digestive neuroendocrine neoplasms (NEN) are a rare and biologically heterogeneous group of tumours. In the past, high-grade (HG) NEN, defined by Ki-67 index >20%, were all classified as neuroendocrine carcinoma. The 2019 WHO classification of digestive tumours separated these tumours into two different disease entities: well-differentiated neuroendocrine tumours with a Ki-67 > 20% (NET G3) and poorly differentiated neuroendocrine carcinomas (NEC) [1]. NET G3 and NEC differ in clinical and molecular characteristics, response to treatment and prognosis [2–6].

Due to few clinical studies on digestive NEC, treatment has been extrapolated from small-cell lung cancer with cisplatin or carboplatin with etoposide as the established first-line palliative chemotherapy [2, 7]. However, the benefit is limited as up to 30% of patients experience immediate disease progression and median overall survival (OS) is only 11-12 months (m) [2, 5, 8–12]. The retrospective NORDIC NEC study showed that cases with a Ki-67 < 55% had a lower response rate (RR) to platinum/etoposide but a longer survival, however this study did not separate between NEC and NET G3 [8]. The optimal treatment for patients with NEC with Ki-67 < 55% remains uncertain. Limited data exist on predictive factors for response to platinum/etoposide treatment in NEC, although some prognostic factors have been identified in retrospective studies [2, 13].

As NET G3 has been defined as late as in 2017 for pancreas and 2019 for the GI-tract, limited data exist. NET G3 seems to constitute 12-18% of all digestive HG-NEN [4–6]. The classification is important as OS for metastatic NET G3 has been reported to be 31–41 m and thus far better than for NEC [4, 14–19]. First- line treatment for metastatic NET G3 has mostly been temozolomide-based regimens or FOLFOX, except for aggressive cases (e.g. cases with Ki-67 ≥ 55%) [4, 15–20]. Peptide receptor radionucleotide therapy (PRRT) was recently established as a treatment option for NET G3 [21, 22]. Data on predictive or prognostic factors for NET G3 are lacking.

NORDIC NEC 2 was a multi-centre prospective observational study collecting clinical data and biological samples from patients with digestive HG-NEN. The cohort was population-based as all patients in the uptake area were treated at the study center, providing real-world data on treatment of this disease. Cases were later pathologically re-classified into NET G3 and NEC. Molecular tumour data on sub-cohorts have been the focus of prior publications [3, 23–25]. Here we present the clinical analysis of the full cohort of 737 patients, constituting the largest study of patients with digestive HG-NEN.

## Methods

Patients diagnosed with HG-NEN with a digestive primary or unknown primary with a predominance of abdominal metastases (digestive primary suspected) were prospectively included during 2013–2017 from nine Scandinavian hospitals providing all oncological treatments of patients with HG-NEN within their region. Clinical data were registered prospectively and transferred to the NORDIC NEC Registry. Cancer pain, weight loss and anorexia were registered at baseline according to National Cancer Institute (NCI) Grading Criteria. Mixed neuroendocrine non-neuroendocrine neoplasms (MiNEN) were included in the registry, but not in the current study. Minimum follow-up time was >5 years. RR was reported according to RECIST v1.1. Patients with reported end of treatment due to clinical progression within 2 months of first course of chemotherapy and without radiological evaluation (all with an early death), were considered to have progressive disease as best response to treatment. Patients were enrolled prior to the formal introduction of NET G3 among digestive HG-NEN [1]. Available tumour sections stained by hematoxylin/eosin, synaptophysin, chromogranin A and Ki-67 were scanned digitally, blinded and re-evaluated according to the 2019 WHO classification in 2021–22 by three experienced NEN pathologists, all three evaluating every case. Initial ambiguous morphological cases were discussed and decided on in a consensus meeting.

### Statistics

Patient and tumour characteristics were analysed using descriptive statistics. Categorical variables were presented as frequences, proportions and percentages, and continuous variables as median/means and ranges, as appropriate. Groups comparisons were performed by *χ*2-test for categorical and Wilcoxon- or Student’s *t*-test for continuous variables. Progression-free survival (PFS) was calculated from the start of first-line chemotherapy to the date of progression, date of death or last known follow-up; OS from start of first-line chemotherapy to date of death or last follow-up. PFS and OS were analysed using the Kaplan-Meier method. Comparisons between groups were performed using the log-rank test or Breslow test. A *p*-value of less than 0.05 was considered significant. Variables with a *p*-value < 0.10 in univariate analyses or assumed of particular potential clinical value were included in multivariate models. Multivariate Cox analysis was done for adjusted models as well as unadjusted models for all predictors in the model. Elevated laboratory values were strongly associated to each other and separate analyses performed to select the most significant laboratory value for the MVA. Analyses were performed using IBM SPSS statistics version 29.0. Figures were created with R software version 4.4.2. Illustrations were created with biorender.com.

## Results

861 patients were prospectively included in the registry. Initial screening of case report forms excluded 58 cases due to other histologic cancer diagnoses than digestive HG-NEN (Fig. S1). Appendix primaries were excluded as few are true HG-NEN and a separation from aggressive goblet cell adenocarcinomas due to nomenclature issues is difficult [26]. Excluding 66 known MiNEN cases resulted in a digestive HG-NEN cohort of 737 cases. Digitalized sections from 498 cases were re-classified centrally. In addition, all remaining cases with a Ki-67 < 60% except six cases, were re-evaluated locally. Pathological re-evaluation excluded another 37 cases (Fig. S1). Most Copenhagen cases (*n* = 145) were not re-classified as all cases had been re-evaluated at study inclusion by a NEN expert pathologist. After reclassification, 700 cases were identified as digestive HG-NEN cases: 564 NEC, 134 NET G3 and two with ambiguous morphology. 119 NEC patients (21%) presented with initially localized disease, 66 had recurrence. 14 NET G3 patients (10%) presented with initially localized disease, eight had recurrence. Twenty-six NET G3 cases (19%) had information of a prior diagnosis of NET G1-2 (23% among pancreatic cases), but none with a family history of NET.

### Patient characteristics and treatment

Main patient characteristics are shown in Table S1/Table 1. Most NEC patients (511/564) had or developed non-resectable advanced disease: 497 metastatic and 14 locally advanced. Almost all NET G3 patients (128/134) had or developed non-resectable advanced disease: 126 metastatic and two locally advanced. Pancreatic primary tumour was seen in 46% of NET G3. Median Ki-67 was 90% for NEC and 30% for NET G3. 12% of NEC and 84% of NET G3 had Ki-67 < 55%. PS 0 was seen in only 23% of NEC. Brain metastases at diagnosis of metastatic disease were rare for NEC (2%) and not seen in NET G3 but evolved in 41 NEC patients (10%) and two NET G3 patients (2%). Almost all patients evaluated by FDG-PET had significant tumour uptake. A high uptake on somatostatin receptor imaging (SRI) was seen for 39% of NEC, although only performed in 1/5 of NEC cases and 1/3 of NEC with an SRI uptake had Ki-67 < 55%. A high uptake on SRI was usually present for NET G3 (80%). Symptom burden at diagnosis of advanced disease was substantial for both NEC and NET G3, and most patients had cancer pain, weight loss and anorexia (Table S2). Reason for death was progressive NEN in the vast majority (>90%), fatal treatment toxicity was registered for six NEC (1.4%) and two NET G3 (2.1%) patients. OS in patients not receiving palliative chemotherapy was 1.9 m for NEC (*n* = 84) and 2.1 m for NET G3 (*n* = 9). Main reasons for not receiving chemotherapy were poor PS (60%), old age (11%) and patient’s choice (11%).

**Table 1:** Selected characteristics of 544 patients with metastatic/advanced digestive HG-NEN treated with chemotherapy (Full details in Table S1).

	NET G3 *n* = 117	NEC *n* = 427	Missing NET G3/NEC
Age median (range)	64 (30–82)	68 (24–89)	
Age >75 years	14 (12%)	88 (21%)	
Male sex	63 (54%)	260 (61%)	
ECOG PS 0	49 (43%)	110 (26%)	3/6
1	49 (43%)	195 (46%)	
2	13 (12%)	92 (22%)	
3	3	23 (5%)	
4	0	1	
Primary tumour			
Esophagus	2	57 (13%)	
Gastric	1	37 (9%)	
Pancreas	54 (46%)	65 (15%)	
Cholangio/gallbladder	0	8 (2%)	
Small intestinal	18 (15%)	8 (2%)	
Colon	8 (7%)	85 (20%)	
Rectum	6 (5%)	79 (19%)	
Unknown abd met^a^	28 (24%)	85 (20%)	
Other	0	3	
Metastatic site			
Liver	113 (97%)	322 (75%)	
Bone	23 (20%)	75 (18%)	
Brain	0	5 (1%)	
SRI uptake ≥ liver	57 (81%)	40 (35%)	47/325
FDG-PET uptake	57 (95%)	150 (98%)	57/274
Ki-67 median	30%	89%	
<55%	100 (85%)	53 (12%)	
≥55%	17 (15%)	374 88%)	
CgA staining Strong	100 (87%)	190 (48%)	2/31
Small-cell morphology		144 (35%)	24 amb
Large-cell morphology		263 (65%)	
ALP elevated	63 (57%)	242(58%)	7/10
LDH elevated	41 (41%)	206 (53%)	18/39

Percentages calculated without missing cases.

*SRI* somatostatin receptor imaging, *ALP* alkaline phosphatase, *LDH* lactate dehydrogenase, *CgA* chromogranin A, *amb* ambiguous, *abd* abdominal.

^a^Suspected digestive primary.

**Fig. 1:**
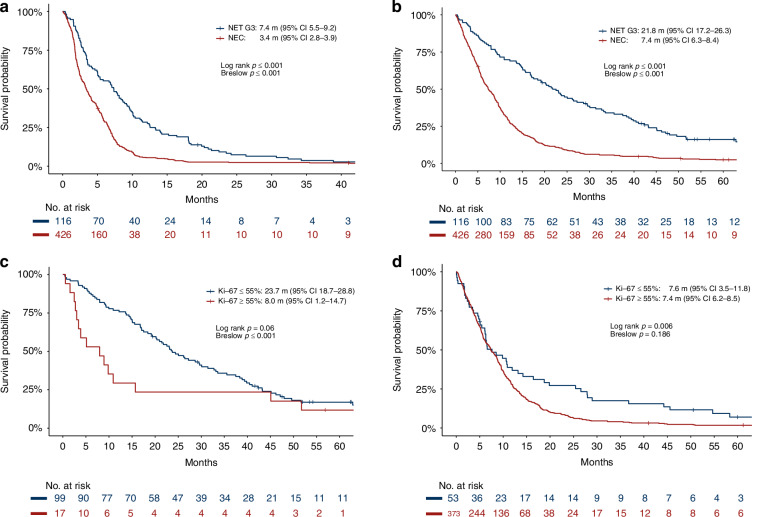
Progression-free survival and overall survival in NET G3 and NEC. Progression-free survival (PFS) (**a**) and overall survival (OS) (**b**) in NET G3 and NEC patients given first-line chemotherapy and OS according to Ki-67 index in NET G3 (**c**) and NEC (**d**).

Palliative first-line chemotherapy was given to 427 NEC (83% platinum/etoposide) and 117 NET G3 (56% temozolomide-based, 38% platinum/etoposide). RR was similar, 31% for NEC and 27% for NET G3. Immediate progression at or prior to first radiological evaluation (PD) was observed in 41% of NEC and 24% of NET G3 (Table 2), corresponding to a disease control rate (DCR) of 59% for NEC and 76% for NET G3. First-line treatment was stopped due to toxicity in 9% for NEC and 13% for NET G3. PFS was twice as long for NET G3 compared to NEC: 7.4 m (95%CI 5.5–9.2) vs 3.4 m (2.8–3.9) (Fig. 1a). OS was three times longer for NET G3 compared to NEC, 21.8 m (17.2–26.3) vs 7.4 m (6.3–8.4) (Fig. 1b). In two cases PFS/OS could not be calculated, one case missing start date of chemotherapy and one case missing PFS/OS date. Three- and five-year survival were 5% and 2% for NEC vs. 32% and 10% for NET G3. A Ki-67 value of 55% separated two prognostic groups within both NEC and NET G3 (Fig. 1c, d). NET G3 patients with Ki-67 ≥ 55% had a similar initial survival as NEC patients (Fig. S2). PFS and OS for patients treated with palliative chemotherapy are shown in Table 2/S4.

**Table 2:** First-line chemotherapy, PFS and OS for NEC and NET G3 patients with advanced disease (See Table S4 for additional results).

	NEC	NET G3
*n*	427	117
Treatment		
Cisplatin/etoposide	61	5
Carboplatin/etoposide	292	40
Temozolomide/capecitabine	19	28
Temozolomide alone	10	9
Temozolomide/everolimus^a^	8	28
Oxaliplatin-based	16	4
Other	21^b^	3
Best response^c^		
CR	8 (2%)	1 (1%)
PR	119 (31%)	28 (26%)
SD	102 (26%)	53 (49%)
PD	158 (41%)	26 (24%)
NA/NE	40	9
PFS	3.4 m (2.8–3.9 m)	7.4 m (5.5–9.2 m)
OS	7.4 m (6.3–8.4 m)	21.8 m (17.2–26.3 m)
OS PS 0	11.2 m (8.9–13.4)	28.7 m (21.5–35.8)
OS PS 1	8.0 m (6.4–9.6)	19.4 m (12.6–26.2)
OS PS 2	5.2 m (4.0–6.4)	5.4 m (0–14.4)
OS PS 3	1.2 m (0.8–1.6)	
OS Ki-67 < 55%	7.6 m (3.5–11.8)	23.7 m (18.7–28.8)
OS Ki-67 ≥ 55%	7.4 m (6.2–8.5)	8.0 m (1.2–14.7)
OS Age < 70	8.3 m (6.8–9.9)	28.7 m (20.1–37.3)
OS Age 70-75	6.4 m (5.0–7.8)	13.6 m (5.5–21.7)
OS Age > 75	6.4 m (4.5–8.3)	17.5 m (5.2–29.8)
OS ALP normal	9.2 m (7.8–10.6)	33.6 m (19.0–48.1)
OS ALP elevated	6.3 m (5.2–7.4)	13.6 m (7.6–19.6)
3-year OS	5%	32%
5-year OS	2%	10%

Median PFS/OS (95%CI).

*NA/NE* not accessed/not evaluable, *PS* performance status, *ALP* alkaline phosphatase.

^a^Phase II study [16].

^b^etoposide mono = 8, capecitabine mono = 5.

^c^RR calculated according to evaluable cases, 5 NET G3 and 47 NEC included as PD with only clinical PD and early death.

### NEC

**Fig. 2:**
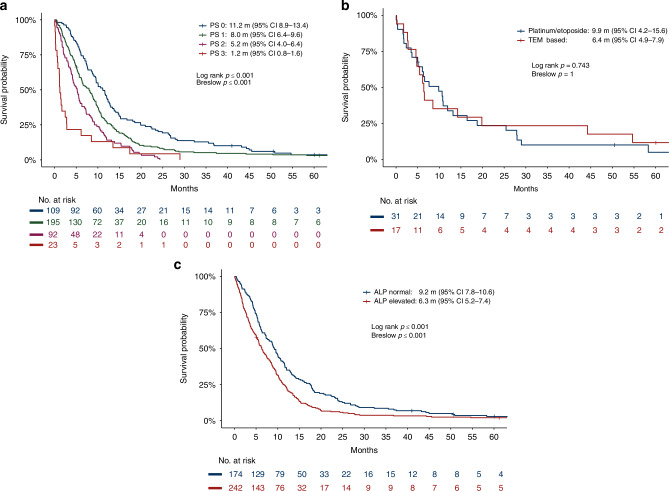
Overall survival in NEC. Overall survival (OS) in NEC patients given first-line chemotherapy according to (**a**) performance status, (**b**) first-line chemotherapy regimen when Ki-67 < 55% and (**c**) alkaline phosphatase (ALP) level.

PS was strongly correlated with no benefit of first-line chemotherapy (immediate progression/PD for PS 0: 29%, PS 2: 49%, PS 3: 71%) and to OS with 11.2 m for PS 0 vs 1.2 m for PS 3 (Fig. 2a). OS was identical comparing platinum/etoposide treatment with other treatments for NEC (Fig. S3). Similar PFS and OS were found comparing NEC patients with Ki-67 < 55% treated with platinum/etoposide vs temozolomide-based regimens (Fig. 2b). Concerning primary tumor site, PFS and OS were shortest for colorectal primaries (2.4 m and 6.7 m) and unknown primaries (3.5 m and 5.8 m), whereas other primary sites had a similar survival (Table S4). Patients with elevated ALP had a shorter survival (Fig. 2c). NEC cases with SRI uptake (*n* = 43) had PFS 4.8 m and OS 13.1 m. OS correlated with response to first-line therapy, 20.6 m (8.9–32.3) after complete response (CR), 11.7 m (10.3-13) for partial response (PR) and 9.9 m (8.6-11.1) for stable disease (SD). Reintroduction of platinum/etoposide was done in 54 NEC patients, response evaluation was available for 47 patients with one CR, 12 PR, 10 SD and 24 PD as best response (RR 28%, DCR 49%). Results of the univariate analyses (UVA) and MVA for PFS and OS are shown in Tables S5–S6. After MVA, significant factors for inferior OS were poor PS, Ki-67 ≥ 55%, elevated ALP, older age and male sex (Table 3). We could not identify a specific age cut-off for inferior survival (Fig. S4). A sensitivity analyses showed no effect of platinum vs non-platinum in the MVA. Significant factors for inferior PFS were poor PS and colorectal or unknown primary site. For later-line treatment see Table S3. Second-line chemotherapy was given to 217 (51%) NEC patients with RR 14%, DCR 31%, PFS 2.1 m (95% CI 1.8–2.3 m) and OS 4.4 m (3.7-5.2 m).

**Table 3:** Final multivariate model for OS and PFS with significant factors (see Tables S5-S8 for full details).

Characteristic	NEC OS *n* = 420			NEC PFS *n* = 420			NET G3 OS *n* = 109			NET G3 PFS *n* = 109		
	HR	95% CI	*p*-value	HR	95% CI	*p*-value	HR	95% CI	*p*-value	HR	95% CI	*p*-value
Age	1,01	1.00, 1.02	0.015				1,05	1.02, 1.08	<0.001			
Female sex	0,79	0.65, 0.98	0.027									
Non-CRC primary	0,83	0.66, 1.04		0,66	0.52, 0.83	0.002						
PS 0	Ref.		<0.001	Ref.		<0.001	Ref.		0.007	Ref.		0.057
1	1,33	1.04, 1.71		1,25	0.98, 1.60		1,45	0.88, 2.39		1,59	0.99, 2.56	
2	1,98	1.45, 2.69		1,6	1.20, 2.15		2,97	1.54, 5.72		1,89	1.02, 3.48	
3	3,73	2.30, 6.03		3,23	2.03, 5.14							
Ki-67 ≥ 55%	1,44	1.05, 1.98	0.02									
ALP > UNL	1,43	1.15, 1.77	0.004				2,74	1.70, 4.43	<0.001			
Platinum/ etoposide treatment							1,83	1.18, 2.84	0.007	3,53	2.14, 5.80	<0.001

*HR* hazard ratio, *CI* confidence interval, *ALP* alkaline phosphatase, *UNL* upper normal limit, *CRC* colorectal, *PS* performance status, *Ref* reference.

### NET G3

**Fig. 3:**
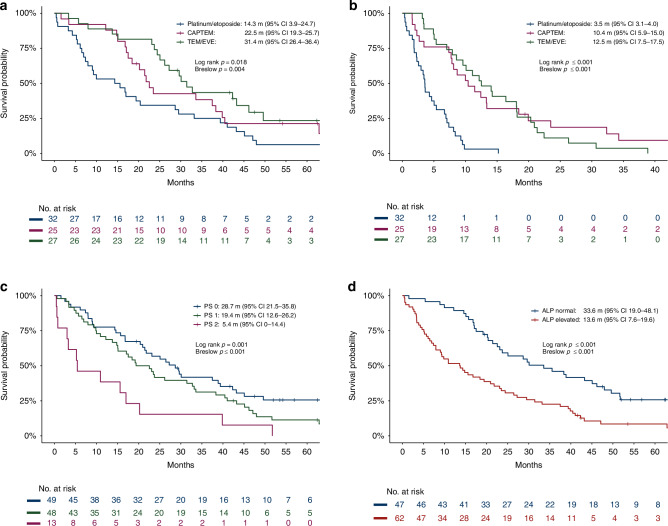
Progression-free survival and overall survival in NET G3. Overall survival (OS) (**a**) and progression-free survival (PFS) (**b**) in NET G3 with Ki-67 < 55% according to first-line chemotherapy regimen and OS in NET G3 according to performance status (**c**) and alkaline phosphatase (ALP) level (**d**).

Patients with NET G3 were further analyzed according to Ki-67 < 55%. For cases with Ki-67 < 55%, first-line platinum-based treatment yielded a much worse outcome than first-line temozolomide-based treatment. PFS and OS for first-line treatment of NET G3 with platinum-etoposide chemotherapy was 3.5 m (95%CI 3.1–4.0) and 14.3 m (3.9–24.7) compared to 10.4 m (5.9–15.0) and 22.5 m (19.3–25.7) with temozolomide/capecitabine and 12.5 m (7.5–17.5) and 31.4 m (26.4–36.4) with temozolomide/everolimus (Fig. 3a, b). UVA and MVA for PFS and OS are shown in Tables S7–S8. After MVA, the significant factors for inferior OS were treatment with platinum/etoposide, poor PS, older age and elevated ALP (Table 3 and Fig. S5). PS and ALP level were strong prognostic factors (Fig. 3c, d). Survival seemed shorter for patients >70 years of age (Fig. S6). For PFS, treatment with platinum/etoposide was significantly inferior and PS borderline significant (*p* = 0.057). Concerning primary tumor site, PFS and OS for pancreatic primaries were 7.7 m and 21.4 m compared to 8.7 m and 37.5 m for small intestinal primaries, however, confidence intervals were overlapping (Table S4). Second-line treatment was given to 78 (67%) NET G3 patients with RR 13%, DCR 67%, PFS 6 m (95% CI 3–9 m) and OS 14 m (4.5–23.4 m). PRRT was given to 27 NET G3 patients (23%), mainly in second-line (*n* = 17), only one in first-line. PFS was 3.7 m (0.9-6.4) and OS 11.1 m (7.3–14.9) from start of second-line chemotherapy, compared to PFS 17.3 m (8.9–25.8) and OS 28.0 m (18.8–37.1) from start of second-line PRRT. PFS was 15.4 m (10–20.8) after PRRT concerning all treatment lines. Overall OS from start of first-line treatment was 46.2 m (41.4–51.0) in patients given PRRT at some time compared to 15.7 m (12.7–18.7) if PRRT was not given.

## Discussion

This large prospective cohort differs from previous published studies as a centralized pathological re-evaluation was done. The study excluded MiNEN and synaptophysin staining adenocarcinoma cases and including only digestive primaries, hence resulting in a large and separate digestive NEC and NET G3 cohort. Furthermore, our analyses were only done on patients with advanced disease. The cohort is population-based as all patients in the uptake area were treated at the study center. It therefore included many elderly patients and patients with poor PS providing real-world data.

### NEC

The primary tumour site was mainly colon, rectum, pancreas and oesophagus, consistent with previous western studies [27]. Few patients had tumours with Ki-67 < 55%, illustrating that this subgroup is infrequent and when found should be re-evaluated concerning a possible NET G3 [14]. A novel observation was that most patients had marked symptomatic disease at diagnosis. Brain metastases were infrequent at diagnosis supporting no role for routine brain imaging [2], however, 10% later developed brain metastasis. In agreement with others [10, 12], almost all cases were positive on FDG-PET. SRI uptake was surprisingly frequent, but SRI was likely performed in selected patients. The main reason for not receiving chemotherapy was poor PS and given the frequent rapid decline in PS seen in these patients, rapid referral for consideration of palliative chemotherapy is important. OS was only 1.9 m for patients not receiving chemotherapy, close to the 1 m seen in NORDIC NEC [8].

Most patients with advanced NEC received first-line treatment with platinum/etoposide. Since NORDIC NEC there has been a strong shift towards using carboplatin rather than cisplatin, due to results showing a similar benefit [8] and better tolerability. Prior studies have shown RR 30–50%, PFS 4–6 m and OS 11–12 m [5, 8–11, 28]. We found a similar RR (34%) but shorter PFS (3.4 m) and OS (7.4 m), likely due to the population-based approach including many patients with poor PS and elderly patients. Looking at our patients with PS 0, OS was 11.2 m. In contrast to other cancers where immediate disease progression on first-line treatment in metastatic disease is rare (<10–15%), we observed primary resistance to therapy in 40% of digestive NEC [5, 8, 10, 28]. There have been attempts to improve first-line treatment, however, two recent Asian randomized trials comparing cisplatin/etoposide vs irinotecan/cisplatin in metastatic digestive NEC (mainly upper GI) found no differences in outcome [11, 28]. Adding nivolumab to first-line platinum/etoposide in a phase II study on digestive NEC led to an OS of 13.9 m [29]. The combination of carboplatin and nab-paclitaxel may be an active first-line treatment for NEC, but this needs further confirmation [30]. Data for FOLFOX or FOLFIRINOX in digestive NEC are limited and restricted to a few case series [31], and results from a phase II trial comparing FOLFIRINOX vs platinum/etoposide (NTC04325425) is awaited. This study should also clarify if NEC should be considered a chemo-resistant disease rather than a platinum/etoposide resistant disease, which will have consequences when exploring new treatment options. Molecular profiling can have the potential to improve patient selection and further studies of molecular factors predicting resistance to chemotherapy are warranted [23, 32]. Our results confirm the lack of progress in treatment for advanced digestive NEC in the last two decades, and even in recent phase II-III studies with selected study patients, OS is still only 11–13 m [11, 28, 29].

Performance status (PS) is an important factor for OS in NEC [8, 10, 13, 14]. Our data shows that chemotherapy should not be given to NEC patients with PS 3 as these patients have a similar poor survival as untreated patients, whereas patients with PS 2 might benefit. In our cohort including a large fraction of elderly, we found a shorter OS with older age which has not been shown previously. We could, however, not identify an age cut-off for shorter survival and the minor numerical difference suggests that high age per se should not preclude patients from chemotherapy. Ki-67 has been shown to be a prognostic factor in some studies [6, 8, 33], however, not in all [10]. In our study Ki-67 < 55% was prognostic and predicted survival in NEC, however, short-time survival was similar. This was in contrast to NET G3 where Ki-67 < 55% had a better short-time survival but similar long-time survival. For NEC with Ki-67 < 55%, our data indicate a similar benefit using platinum/etoposide vs temozolomide-based treatment. However, numbers were limited and patients not randomized, so the optimal treatment is still to be determined for this subgroup. Female sex was a favorable prognostic factor. Previous studies have shown a similar general gender-related OS difference in NEN [34–36] as in most other cancer types for unknown reasons. Elevated ALP and lactate dehydrogenase (LDH) have been reported to be prognostic factors for shorter OS in metastatic NEC [8, 9, 13, 14, 37]. In the present cohort elevated levels of ALP, LDH, CRP and leucocytes had prognostic value, however, ALP was the strongest factor and significant in the MVA. Little is known about factors affecting PFS in digestive NEC. We found that colorectal primary predicted for short PFS, however, not for OS suggesting that colorectal NEC are relatively resistant to carboplatin/etoposide but may respond to subsequent therapy. Similar findings were presented in another cohort where colon NEC had a significantly shorter PFS compared to other primary digestive sites but with no effect on OS and independent of *BRAF* mutation status [32]. Taken together it must be questioned if platinum/etoposide should be the preferred first-line treatment for colorectal NEC.

### NET G3

Our study adds significantly to the limited prior data on NET G3. NET G3 represented 20% of high-grade digestive NEN, previous studies report an incidence of 10-20% [4–6, 38]. Most NET G3 primaries were pancreatic. Pancreatic NET G3 was almost as frequent as pancreatic NEC among high-grade pancreatic NEN cases, which is important to have in mind at diagnosis of a high-grade pancreatic NEN [14, 39]. 90% of NET G3 presented with metastatic disease. Patients with metastatic disease were usually symptomatic with cancer pain, weight loss or anorexia. Median Ki-67 was 30% as in prior studies [4], only 16% had Ki-67 > 55%. Almost all NET G3 had FDG-PET uptake. SRI uptake was seen in 80% of cases, showing that PRRT could be a treatment option for most NET G3 [21, 22]. Treatment for metastatic NET G3 varied in our cohort. As NET G3 was not an established disease entity at the time of the study, many cases were treated as having NEC. Patients were mainly treated with temozolomide-based treatment or platinum/etoposide. This provides a unique opportunity to compare results of first-line treatments for NET G3 with Ki-67 < 55%. First-line platinum/etoposide to NET G3 with Ki-67 < 55% leads to a much shorter PFS and OS compared to temozolomide-based treatment. Our study shows that when using first-line chemotherapy for NET G3 with Ki-67 < 55%, the best chemotherapy schedule must be used initially as later lines of treatments could not compensate for sub-optimal first-line chemotherapy. This emphasizes the importance of the initial pathological work-up separating NET G3 from NEC. A specialist NET pathologist review is crucial to ensure all patients tumors get an optimal pathology review, however, separating NET G3 from NEC can be difficult even among NET experts [14]. Our study confirms prior studies indicating that platinum/etoposide is an inferior treatment for NET G3 [5, 14, 17, 19]. In our cohort, PFS and OS after temozolomide-based treatment seems shorter than in prior retrospective studies [15, 17–19, 38], likely due to its population-based inclusion of elderly patients and patients with worse prognostic factors such as PS. The NETTER-2 trial demonstrated major PFS superiority of PRRT versus somatostatin analog therapy as first-line treatment for the NET G3 subgroup [40]. PRRT can now be considered as a potential first-line treatment for somatostatin receptor positive NET G3 patients, but whether it should be the first-line standard of care for all NET G3 patients with Ki-67 < 55% is still not clarified [22]. The overall PFS (15.4 m) and OS (46.2 m) for NET G3 patients receiving PRRT in our study supports PRRT as an important treatment option for NET G3.

Few if any prognostic factors have previously been identified for NET G3. We found that in addition to platinum-based treatment, significant factors for short OS were poor PS, older age and elevated ALP. The OS for patients with PS 2 was surprisingly low, only 5.4 m and similar to NEC PS 2 patients. Survival was shorter in elderly patients, however OS was still 17.5 m for patients above 75 years indicating benefit of treatment for elderly patients. The impact of elevated ALP on treatment outcome for NET G3 was strikingly large and patients with elevated ALP might be a subgroup needing a different treatment strategy. The few cases of NET G3 with a Ki-67 ≥ 55% had a short OS resembling NEC, which supports that the Ki-67 value seems more important than morphology when considering treatment for these cases [20]. The NETTER-2 trial included only NET G3 with Ki-67 < 55% [21], and PRRT should probably not be given as first-line treatment for NET G3 with a Ki-67 ≥ 55% [22]. The three- and five-year survival rates of 32% and 10% seem marked lower than for NET G2 [41, 42], highlighting the more aggressive nature of NET G3.

### Limitations

This is a large digestive HG-NEN cohort however some subgroups have limited numbers. As disease characteristics and treatment might differ according to primary site, further data on different primary digestive sites are needed. Patients were included on a population basis however it is not known how many eligible patients were not included. Treatment and inclusion of patients were based on old pathological classifications. Results of comparisons according to treatment strata should be interpreted with care as patients were not randomized and selection bias cannot be excluded. Predictive or prognostic molecular factors were not analyzed in this study.

## Conclusions

This large prospective population-based multicenter study adds a substantial amount of new data for advanced digestive NEC and NET G3, relevant for the clinicians when selecting treatment and considering the prognosis for the patient. Our study highlights the clinical differences between NET G3 and NEC, emphasizing the importance of differentiating NET G3 from NEC during the initial pathological work-up. Treating NET G3 as NEC initially leads to a poorer survival outcome. The short OS of 7.4 m and 3-year survival of 5% demonstrate that metastatic digestive NEC is one of the most aggressive cancers with an unmet need for improved treatment.

## Supplementary information

Supplementary Figures 1-6

Supplementary Tables 1-8

## Data Availability

All relevant data related to this study are included within the article or in the supplementary materials. Further data will be provided upon reasonable request to the corresponding author.
