# Correction: Characteristics and treatment outcome in a prospective cohort of 639 advanced high-grade digestive neuroendocrine neoplasms (NET G3 and NEC). The NORDIC NEC 2 study

**DOI:** 10.1038/s41416-025-03095-1

**Published:** 2025-07-14

**Authors:** Halfdan Sorbye, Geir Olav Hjortland, Lene Weber Vestermark, Morten Ladekarl, Johanna Svensson, Anna Sundlöv, Eva Tiensuu Janson, Herish Garresori, Eva Hofsli, Christian Kersten, Hege Elvebakken, Per Pfeiffer, Siren Morken, Jorg Assmus, Inger Marie Bowitz Lothe, Elizaveta Tabaksblat, Ulrich Knigge, Anne Couvelard, Aurel Perren, Seppo W. Langer

**Affiliations:** 1 Cancer Clinic, Haukeland University Hospital, Bergen, Norway; 2 Department of Clinical Science, University of Bergen, Bergen, Norway; 3 Department of Oncology, Oslo University Hospital, Oslo, Norway; 4 Department of Oncology, University Hospital of Southern Denmark, Esbjerg, Denmark; 5 Department of Oncology, Aarhus University Hospital, Aarhus, Denmark; 6 Department of Oncology, Clinical Cancer Research Center, Aalborg University Hospital, and Department of Clinical Medicine, Aalborg University, Aalborg, Denmark; 7 Department of Oncology, Sahlgrenska University Hospital, Gothenburg, Sweden; 8 Department of Oncology, Skåne University Hospital, Lund, Sweden; 9 Department of Medical Sciences, Section of Endocrine Oncology, Uppsala University, Uppsala, Sweden; 10 Department of Oncology, Stavanger University Hospital, Stavanger, Norway; 11 Department of Oncology, St.Olavs Hospital, Trondheim, Norway; 12 Department of Research, Hospital of Southern Norway, Kristiansand, Norway; 13 Department of Oncology, Ålesund Hospital, Møre og Romsdal Hospital Trust, Ålesund, Norway; 14 Department of Oncology, Odense University Hospital, Odense, Denmark; 15 Centre for Clinical Research, Haukeland University Hospital, Bergen, Norway; 16 Department of Pathology, Oslo University Hospital, Oslo, Norway; 17 Department of Surgery C and Endocrinology PE, Rigshospitalet, Faculty of Health Science, University of Copenhagen, Copenhagen, Denmark; 18 Department of Pathology, Bichat Hospital, Paris, France; 19 Institute of Tissue Medicine and Pathology, University of Bern, Bern, Switzerland; 20 Department of Oncology, Rigshospitalet, Copenhagen, Denmark; 21 Department of Clinical Medicine, University of Copenhagen, Copenhagen, Denmark

**Keywords:** Outcomes research, Neuroendocrine cancer

Correction to: *British Journal of Cancer* 10.1038/s41416-025-03054-w, published online 17 May 2025

Following publication of the article, an error was found in the descriptive text for Table 2; the article originally stated “Median PFS/OS (range)”, but the correct description is “Median PFS/OS (95% CI)”.

The original article has been corrected; the correction does to affect the results or conclusions or the article.

