# Supplementary material for: Characteristics and treatment outcome in a prospective cohort of 639 advanced high-grade digestive neuroendocrine neoplasms (NET G3 and NEC). The NORDIC NEC 2 study
Source: Br J Cancer. 2025 May 17;133(3):316–24. doi: 10.1038/s41416-025-03054-w (PMC12322073; doi:10.1038/s41416-025-03054-w)

Figure S1. Flow digram of case selection and exclusion reasons.

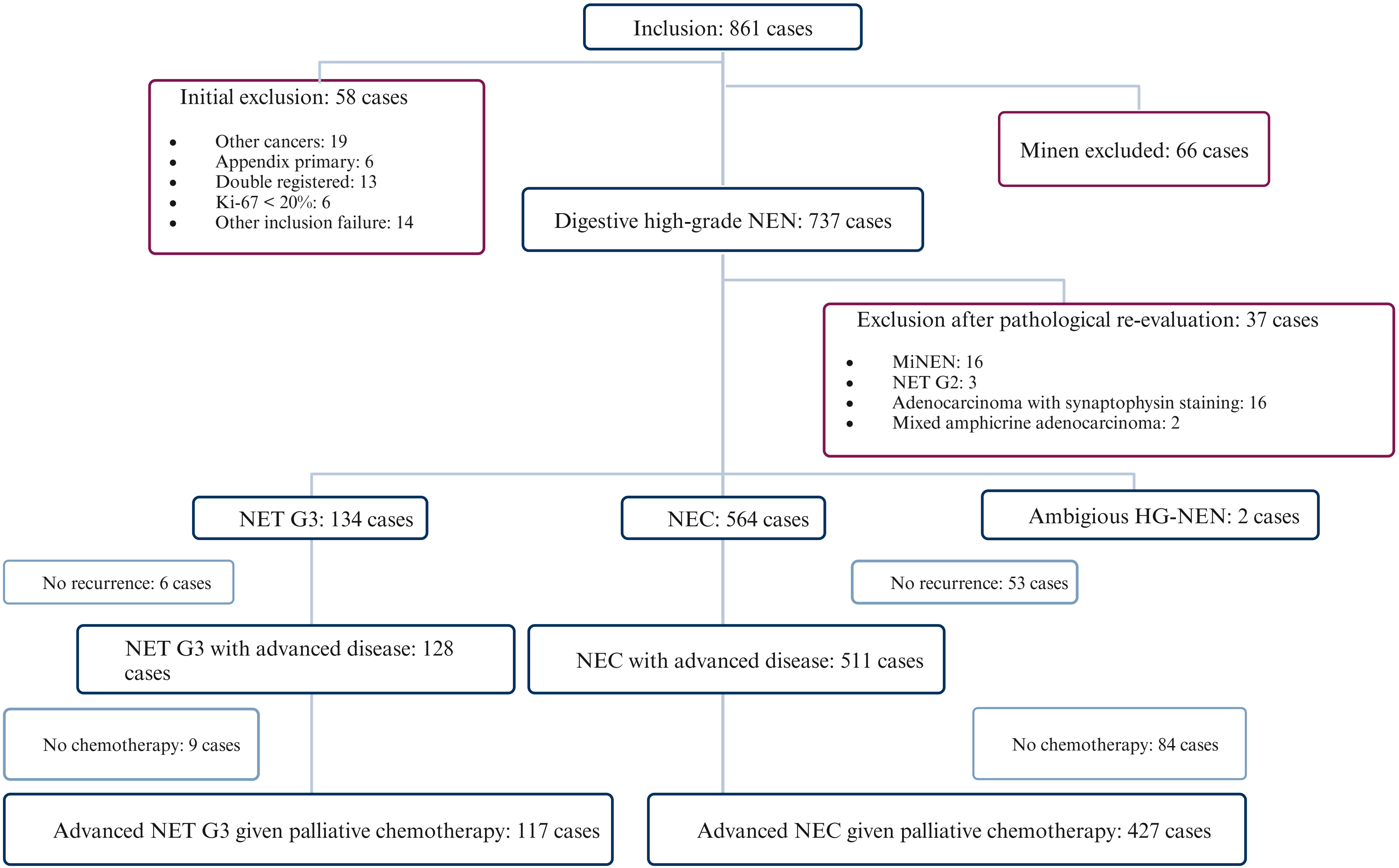

Figure S2. OS in NEC and NET G3 given first−line chemotherapy according to Ki−67 55% cutoff

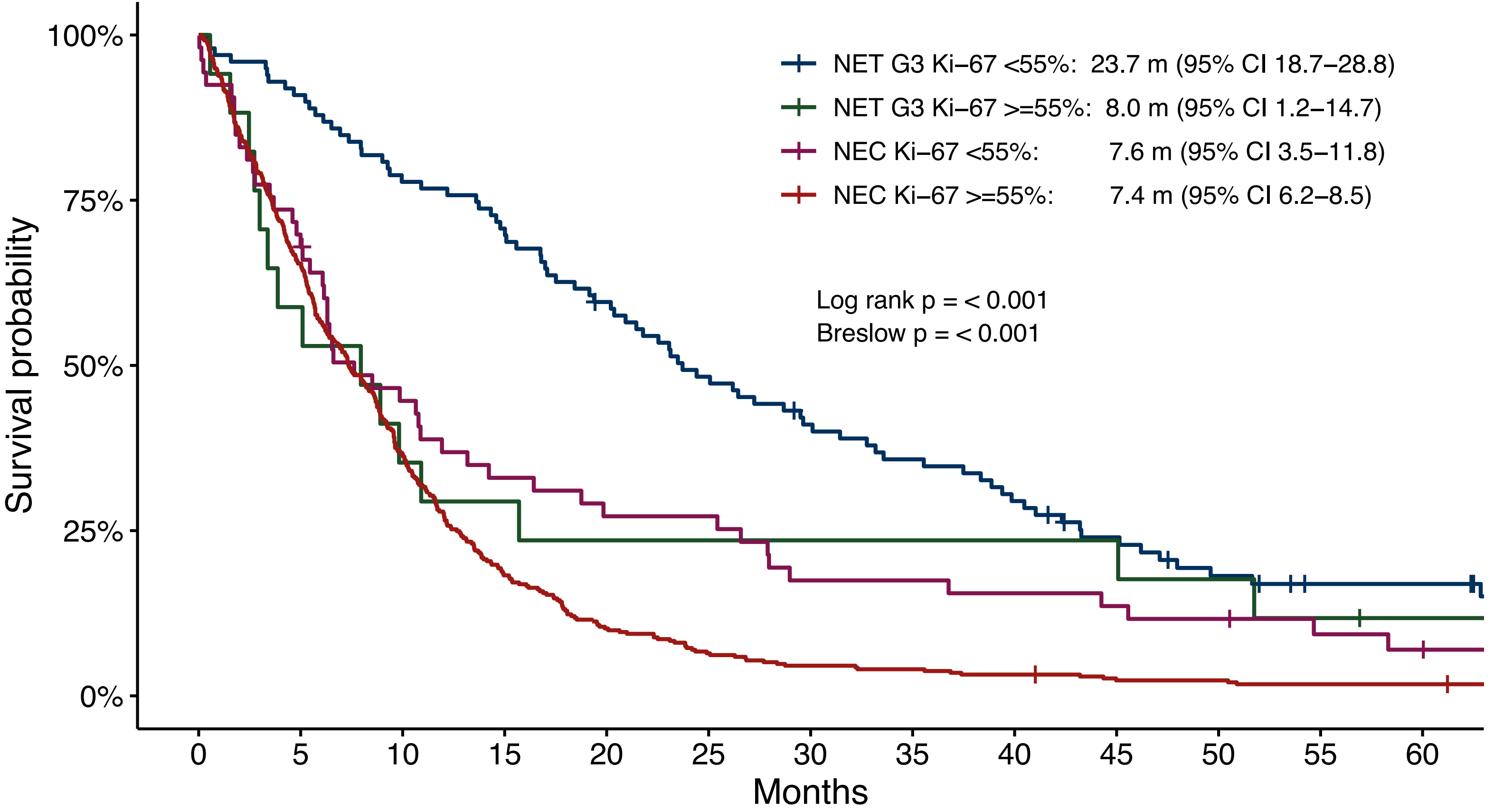

No. at risk

NET G3 Ki−67 <55%	99	90	77	70	58	47	39	34	28	21	15	11	11
NET G3 Ki−67 >=55%	17	10	6	5	4	4	4	4	4	4	3	2	1
NEC Ki−67 <55%	53	36	23	17	14	14	9	9	8	7	6	4	3
NEC Ki−67 >=55%	373	244	136	68	38	24	17	15	12	8	8	6	6

Figure S3. OS in NEC given first–line chemotherapy according to regimen

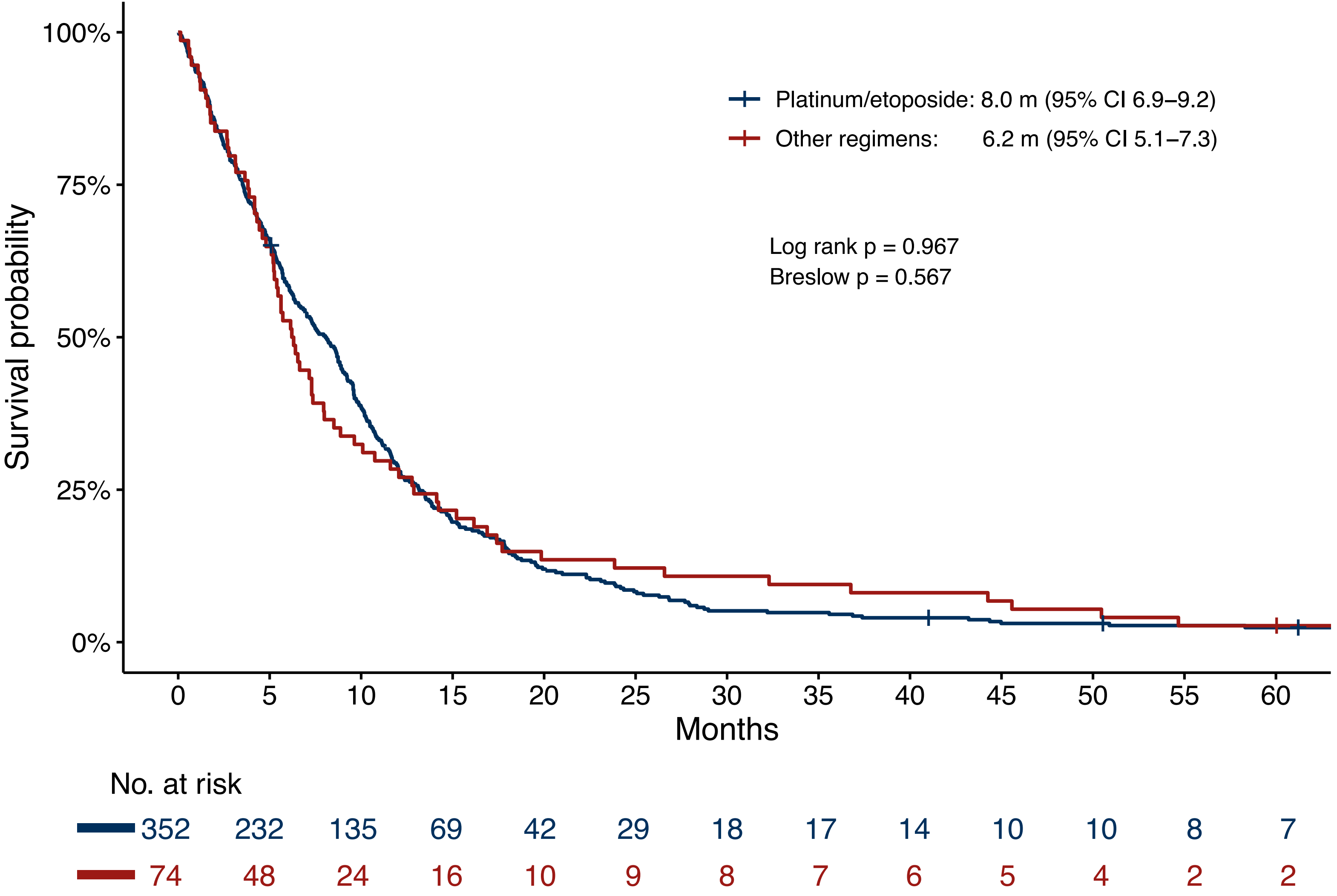

Figure S4. OS in NEC given first−line chemotherapy according to age

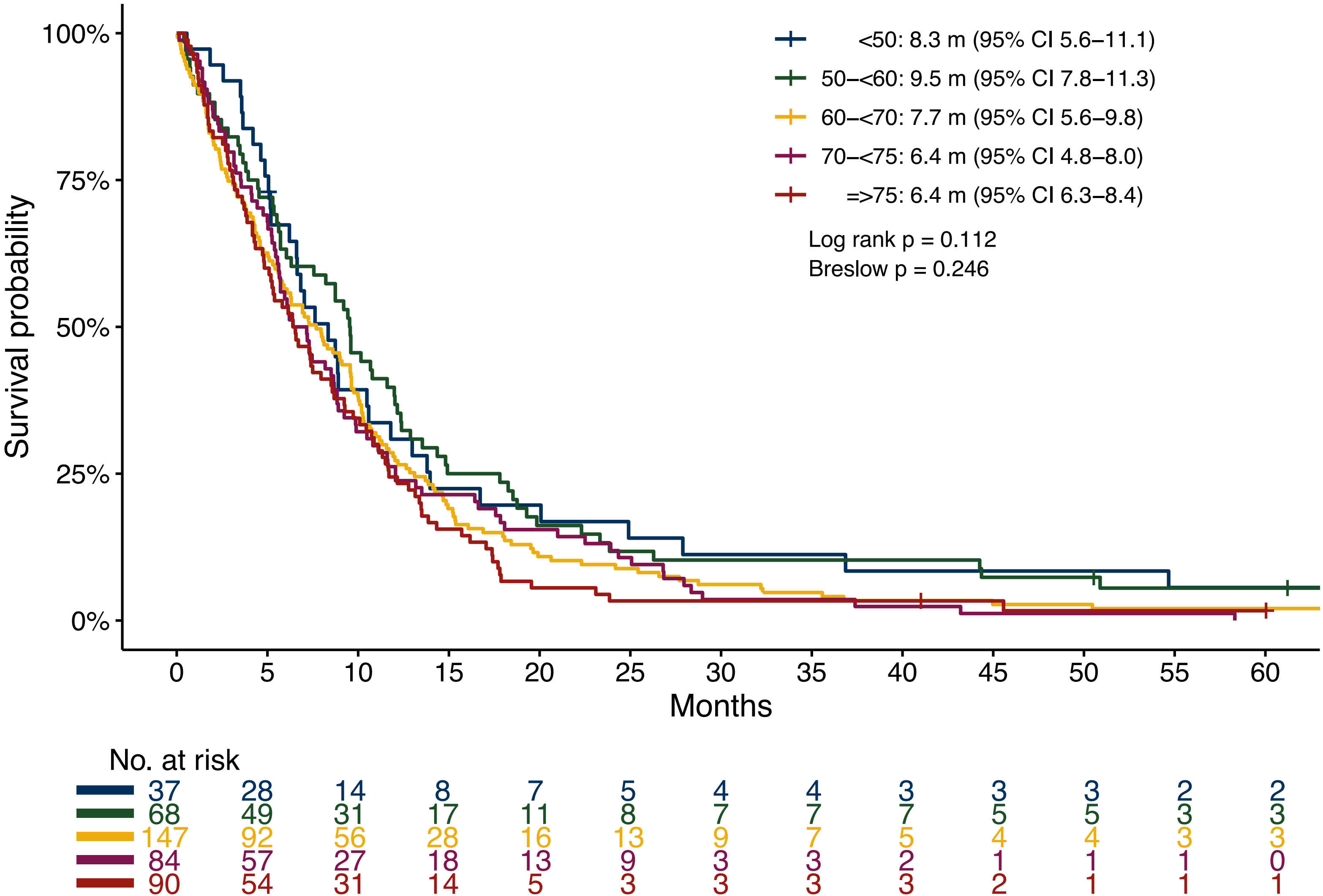

Figure S5. Significant prognostic factors for survival after multivariate analysis.

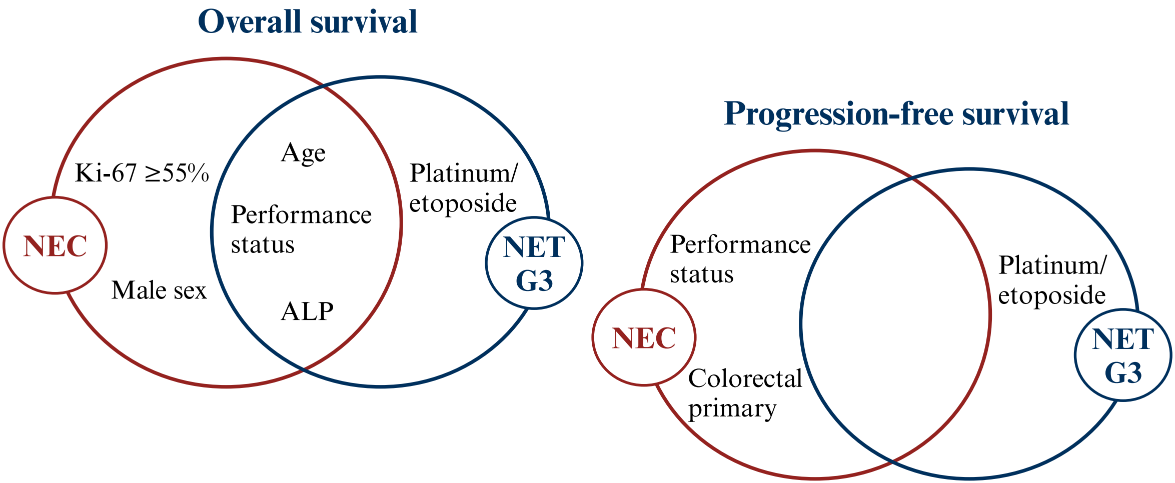

Figure S6. OS in NET G3 given first–line chemotherapy according to age

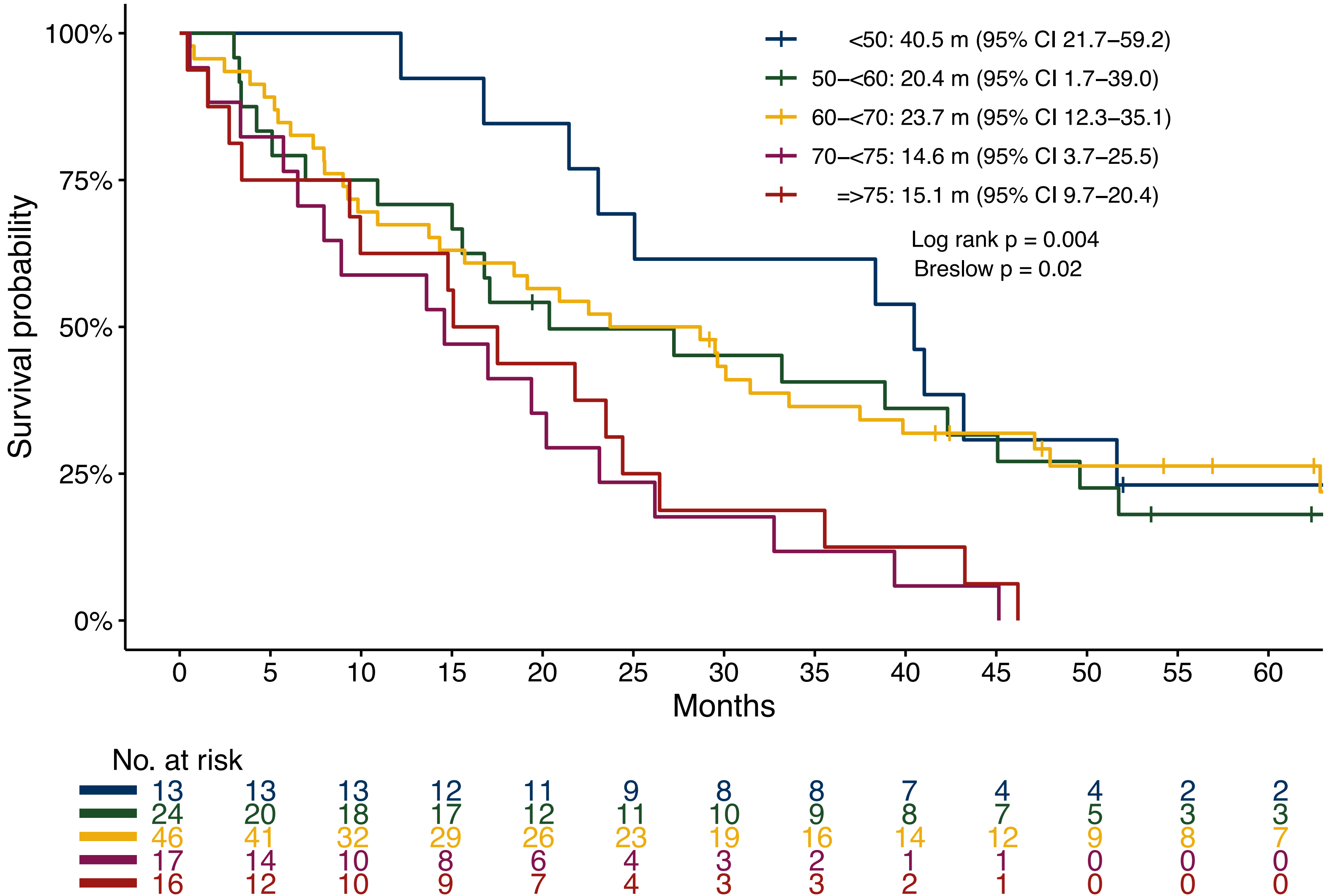

Supplement: Supplementary file 1 — Supplementary Figures 1-6 [file 41416_2025_3054_MOESM1_ESM.pdf]
