# Supplementary material for: Characteristics and treatment outcome in a prospective cohort of 639 advanced high-grade digestive neuroendocrine neoplasms (NET G3 and NEC). The NORDIC NEC 2 study
Source: Br J Cancer. 2025 May 17;133(3):316–24. doi: 10.1038/s41416-025-03054-w (PMC12322073; doi:10.1038/s41416-025-03054-w)
Supplement: Supplementary file 2 — Supplementary Tables 1-8 [file 41416_2025_3054_MOESM2_ESM.pdf]

Table S1. Characteristics of 639 patients with metastatic/advanced HG-digestive NEN, 544 treated with chemotherapy.

	All NET G3 n= 128	All NEC n= 511	All missing	Chemotherapy treated NET G3 n=117	Chemotherapy treated NEC n=427
Age median (range)	66 (30-90)	69 (24-93)		64 (30-82)	68 (24-89)
Age > 75 years	18	134		14 (12%)	89 (21%)
Male sex	69 (54%)	310 (61%)		63 (54%)	260 (61%)
ECOG PS 0	54 (43%)	116 (23%)	12	49 (43%)	110 (26%)
1	49 (40%)	214 (43%)		49 (43%)	195 (46%)
2	14 (11%)	107 (21%)		13 (12%)	92 (22%)
3	6 (5%)	50 (10%)		3 (3%)	23 (5%)
4	1	15 (3%)		0	1
Primary tumor					
Esophagus	3 (2%)	69 (14%)		2	57 (13%)
Gastric	1	46 (9%)		1	37 (9%)
Pancreas	59 (46%)	76 (15%)		54 (46%)	65 (15%)
Cholangio/gallbladder	1	8 (2%)		0	8 (2%)
Small intestinal	19 (15%)	10 (2%)		18 (15%)	8 (2%)
Colon	8 (6%)	103 (20%)		8 (7%)	85 (20%)
Rectum	7 (5%)	93 (18%)		6 (5%)	79 (19%)
Unknown abd met*	30 (23%)	103 (20%)		28 (24%)	85 (20%)
Other	0	3		0	3
Primary tumor resected	31(24%)	113 (22%)	1	28 (24%)	89 (21%)
Metastatic site					
Liver	122 (95%)	378 (74%)		113 (97%)	322 (75%)
Lung	18 (14%)	95 (19%)		14 (12%)	84 (20%)
Distant lymph nodes	37 (29%)	165 (32%)		36 (31%)	151 (35%)
Bone	24 (19%)	87 (17%)		23 (20%)	75 (18%)
Brain	0	9 (2%)		0	5 (1%)
Other	20 (16%)	65 (13%)		17 (15%)	54 (13%)
Smoker/prior smoker	57 (49%)	279 (60%)	58	53/71 (49%)	234/392 (60%)
Somatostatin receptor imaging, uptake >= liver	58/73 (80%)	43/111 (39%)	55 NET G3 400 NEC	57/70 (81%)	40/102 (35%)
FDG-PET uptake	60/63 (95%)	170/173 (98%)	65 NET G3 338 NEC	57/60 (95%)	150/153 (98%)
Ki-67 mean	39 %	81 %		38%	80%
median	30 %	90 %		30%	89%
< 55%	107 (84%)	63 (12%)		100 (85%)	53 (12%)
≥55%	21 (16%)	448 (88%)		17 (15%)	374 88%)
CgA staining Absent	6 (5%)	151 (32%)	40	6 (5%)	119 (30%)
Weak/partial	12 (10%)	104 (22%)		9 (8%)	87 (22%)
Strong	107 (86%)	219 (46%)		100 (87%)	190 (48%)
Small-cell morphology		163 (33%)	24 amb		144 (35%)
Large-cell morphology		324 (67%)	24 amb		263 (65%)
5-HIAA elevated	16/30	20/80	529	15/28	19/73

ALP elevated	71/120 (59%)	282/487 (58%)	32	63/110 (57%)	242/417 (58%)
LDH elevated	44/104 (42%)	237/445 (53%)	88	41/99 (41%)	206/388 (53%)
Platelets > 400 x 10 9/L	15/124 (12%)	135/510 (26%)	24	13/113 (12%)	99/426 (23%)
WBC > 10 x 10 9/L	28/124 (23%)	188/510 (37%)	25	23/113 (20%)	142/426 (33%)
CRP > 10	47/103 (46%)	261/415 (63%)	121	41/95 (43%)	209/349 (60%)
NSE elevated	41/56 (73%)	176/227 (78%)	356	38/52 (73%)	157/204 (77%)
CgA elevated	84/107 (79%)	222/355 (63%)	175	80/103(78%)	190/318 (60%)

Percentages calculated without missing cases. ALP= alkaline phosphatase, LDH= lactate dehydrogenase, WBC= white blood count, NSE= neuron specific enolase, CgA= chromogranin A, amb= ambiguous, abd= abdominal. met= metastases. *Suspected digestive primary.

Table S2. Symptom burden at diagnosis of metastatic disease (609/639 available) according to National Cancer Institute (NCI) Grading Criteria.

		All grades	NCI grade 1	NCI grade 2	NCI grade 3
NEC	Cancer pain	66%	33%	25%	8%
	Weight loss	54%	28%	23%	3%
	Anorexia	58%	36%	17%	4%
NET G3	Cancer pain	63%	41%	15%	6%
	Weight loss	45%	23%	21%	1%
	Anorexia	59%	41%	15%	3%

Table S3. Later-line treatment for NEC (n=427) and NET G3 (n=117) patients.

	NEC	NET G3
Second-line treatment	217 (51%)	78 (67%)
Third-line treatment	83 (19%)	35 (30%)
Fourth-line treatment	22 (5%)	17 (15%)
Secondary metastatic surgery	17 (4%)	9 (8%)
PRRT	5 (1%)	27 (23%)
Palliative radiotherapy	133 (31%)	22 (19%)

Table S4. OS and PFS for NEC and NET G3 patients given first-line palliative chemotherapy.

	NEC		NET G3	
	OS (95%CI)	PFS (95%CI)	OS (95%CI)	PFS (95%CI)
ECOG PS				
0	11.2 m (8.9-13.4)	4.6 m (3.6-5.7)	28.7 m (21.5-35.8)	8.7 m (6.8-10.5)
1	8.0 m (6.4-9.6)	3.4 m (2.7-4.1)	19.3 m (12.6-26.2)	6.9 m (3.7-10.1)
2	5.2 m (4.0-6.4)	2.6 m (1.6-3.6)	5.4 m (0-14.4)	3.5 m (2.8-4.2)
3	1.2 m (0.8-1.6)	1.2 m (0.9-1.5)		
Ki-67				
< 55%	7.6 m (3.5-11.8)	2.7 m (2.1-3.2)	23.7 m (18.7-28.8)	7.9 m (6.5-9.2)
≥55%	7.4 m (6.2-8.5)	3.5 m (3.0-4.0)	8.0 m (1.2-14.7)	5.0 m (2.1-7.9)
Primary site				
oesophageal	9.0 m (6.8-11.2)	4.9 m (2.8-6.9)		
gastric	8.1 m (6.1-10.2)	3.7 m (1.8-5.6)		
pancreas	8.9 m (5.7-12.0)	4.8 m (3.4-6.2)	21.4 m (15.6-27.3)	7.7 m (6.1-9.4)
small intestine	8.5 m (3.6-13.4)	4.1 m (1.7-6.6)	37.5 m (19.3-55.7)	8.7 m (4.9-12.5)
colorectal	6.7 m (5.6-8.8)	2.4 m (2.1-3.3)		
unknown	5.8 m (4.1-7.5)	3.5 m (2.5-4.4)	18.4 m (5.7-31.1)	7.4 m (2.6-12.4)
Age				
<50	8.3 m (5.6-11.1)	4.1 m (1.6-6.6)	38.3 m (10.1-66.6)	7.7 m (6.3-9.1)
50-60	9.5 m (7.8-11.2)	3.4 m (2.2-4.6)	27.2 m (3.7-50.8)	7.1 m (1.4-12.8)
60-70	7.7 m (5.6-9.8)	3.1 m (2.2-3.9)	23.7 m (12.3-35.1)	7.3 m (5.3-9.3)
70-75	6.4 m (5.0-7.8)	3.7 m (2.3-5.1)	13.6 m (5.5-21.7)	5.0 m (2.2-7.8)
>75	6.4 m (4.5-8.3)	2.6 m (1.8-3.5)	17.5 m (5.2-29.8)	5.4 m (0.9-9.8)
SRI Uptake				
None or <Liver	9.6 m (6.7-12.5)	4.0 m (1.9-6.1)	17.1 m (10.8-23.3)	5.4 m (0.2-10.6)
>Liver	13.1 m (3.9- 22.3)	4.8 m (2,7-6.9)	30.1 m (21.7-38.5)	9.5 m (7.3-11.7)
Blood values				
WBC normal	8.8 m (7.7-10.0)	3.5 m (2.9-4.1)	23.7 m (17.4-30.0)	7.9 m (5.9-10.0)
elevated	5.3 m (4.6-6.1)	3.2 m (2.3-4.0)	9.0 m (0.8-17.2)	4.6 m (2.1-7.1)
Small-cell	8.9 m (6.8-11)	4.8 m (3.5-6.1)		
Large cell	7.5 m (6.1-8.8)	3.2 m (2.5-3.8)		
ALP normal	9.2 m (7.8-10.6)	3.4 m (2.6-4.4)	33.6 m (19.0-48.1)	8.6 m (5.0-12.1)
elevated	6.3 m (5.2-7.4)	3.3 m (2.6-3.9)	13.6 m (7.6-19.6)	5.0 m (1.4-8.6)
LDH normal	10.1 m (8.9-11.2)	3.8 m (3.1-4.5)	22.5 m (18.2-26.9)	7.9 m (6.0-9.7)
elevated	5.9 m (5.3-6.5)	2.8 m (2.1-3.6)	15.7 m (0.8-30.6)	4.6 m (0.8-8.5)
CRP < 10	9.9 m (8.9-10.8)	4.1 m (2.9-5.3)	30.1 m (21.4-38.8)	10.0 m (7.9-12.1)
> 10	6.1 m (5.0-7.3)	3.1 m (2.5-3.9)	9.8 m (6.1-13.5)	4.3 m (2.4-6.1)

Table S5. Univariate and multivariate analyses for PFS in NEC.

	Univariable				Full model				Blood variables				Final model			
**Characteristic**	**N**	**HR**	**95% CI**	**p-value**	**N**	**HR**	**95% CI**	**p-value**	**N**	**HR**	**95% CI**	**p-value**	**N**	**HR**	**95% CI**	**p-value**
Age	426	1,01	1.00, 1.02	**0.039**	343	1,01	1.00, 1.02	0.258	349	1,01	1.00, 1.02	**0.061**	420	1	1.00, 1.01	0.337
Sex	426			0.629	343			0.121	349			0.408	420			0.171
Male		Ref.	—			Ref.	—			Ref.	—			Ref.	—	
Female		0,95	0.78, 1.16			0,83	0.66, 1.05			0,91	0.73, 1.14			0,87	0.71, 1.06	
Primary tumor	426			**0.014**	343			**0.011**					420			**0.002**
colorectal		Ref.	—			Ref.	—							Ref.	—	
CUP		0,97	0.74, 1.27			0,83	0.60, 1.15							0,79	0.60, 1.05	
other		0,74	0.60, 0.92			0,67	0.51, 0.87							0,66	0.52, 0.83	
PS	420			**<0.001**	343			**0.03**					420			**<0.001**
0		Ref.	—			Ref.	—							Ref.	—	
1		1,2	0.95, 1.52			1,12	0.85, 1.49							1,25	0.98, 1.60	
2		1,49	1.12, 1.97			1,39	0.98, 1.98							1,6	1.20, 2.15	
3		2,86	1.84, 4.47			2,3	1.31, 4.02							3,23	2.03, 5.14	
Ki-67 cont.	426	1	0.99, 1.00	0.404	343	0,99	0.98, 1.00	0.116					420	1	0.99, 1.00	0.057
Ki-67	426			0.712	343			0.285								
Ki-67 <55%		Ref.	—			Ref.	—									
Ki-67 ≥55%		0,95	0.70, 1.27			1,35	0.78, 2.36									
CRP cont.	349	1	1.00, 1.00	**0.015**	343	1	1.00, 1.00	0.374	349	1	1.00, 1.00	0.128				
CRP	349			**0.045**	343			0.693								
<10		Ref.	—			Ref.	—									
≥10		1,25	1.00, 1.55			1,06	0.80, 1.39									
CgA	424			0.858	343			0.959								
normal		Ref.	—			Ref.	—									
high		1,01	0.80, 1.27			1,03	0.78, 1.37									
not done		1,07	0.82, 1.39			1	0.71, 1.39									
LDH	425			0.329	343			0.846	349			0.934				
normal		Ref.	—			Ref.	—			Ref.	—					
high		1,16	0.95, 1.42			1,05	0.81, 1.37			1,05	0.82, 1.34					
not done		1,02	0.72, 1.45			0,93	0.59, 1.47			1	0.65, 1.53					
ALP	425			0.387	343			0.979	349			0.695				
normal		Ref.	—			Ref.	—			Ref.	—					
high		1,11	0.91, 1.35			1,02	0.80, 1.31			1,11	0.87, 1.40					
not done		0,79	0.40, 1.54			0,95	0.37, 2.43			1,13	0.45, 2.89					
Platelets	425			0.857	343			0.6	349			0.416				
normal		Ref.	—			Ref.	—			Ref.	—					
high		1,07	0.85, 1.35			0,95	0.72, 1.27			0,88	0.67, 1.16					
not done		0,97	0.36, 2.61			3,2	0.41, 24.7			3,21	0.43, 24.2					
Leucocytes	425			**0.061**	343			0.768	349			0.366				
normal		Ref.	—			Ref.	—			Ref.	—					
high		1,23	1.00, 1.52			1,08	0.81, 1.43			1,14	0.86, 1.50					
not done		0,66	0.31, 1.40			0,68	0.15, 3.19			0,46	0.10, 2.16					

HR = Hazard Ratio, CI = Confidence Interval. CUP= unknown primary PS= performance status. cont= continous, CgA=chromogranin A staining, LDH=lactate dehydrogenase, ALP=alkaline phosphatase

Table S6. Univariate and multivariate analyses for OS in NEC.

	Univariable				Full model				Blood variables				Final model			
**Characteristic**	**N**	**HR**	**95% CI**	**p-value**	**N**	**HR**	**95% CI**	**p-value**	**N**	**HR**	**95% CI**	**p-value**	**N**	**HR**	**95% CI**	**p-value**
Age	426	1,01	1.00, 1.02	**0.002**	343	1,01	1.00, 1.02	**0.012**	349	1,02	1.01, 1.03	**<0.001**	420	1,01	1.00, 1.02	**0.015**
Sex	426			0.481	343			**0.01**	349			**0.07**	420			**0.027**
Male		Ref.	—			Ref.	—			Ref.	—			Ref.	—	
Female		0,93	0.76, 1.14			0,73	0.57, 0.93			0,81	0.65, 1.02			0,79	0.65, 0.98	
Primary tumor	426			**0.042**	343			0.362					420			0.217
colorectal		Ref.	—			Ref.	—							Ref.	—	
CUP		1,21	0.92, 1.58			0,96	0.69, 1.34							0,96	0.73, 1.28	
other		0,86	0.69, 1.07			0,83	0.64, 1.09							0,83	0.66, 1.04	
PS	420			**<0.001**	343			**<0.001**					420			**<0.001**
0		Ref.	—			Ref.	—							Ref.	—	
1		1,46	1.15, 1.86			1,18	0.89, 1.56							1,33	1.04, 1.71	
2		2,3	1.73, 3.06			1,89	1.32, 2.71							1,98	1.45, 2.69	
3		4,03	2.58, 6.31			2,52	1.43, 4.43							3,73	2.30, 6.03	
Ki-67 cont	426	1,01	1.00, 1.01	**0.027**	343	1	0.99, 1.01	0.904								
Ki-67	426			**0.004**	343			0.243					420			**0.02**
Ki-67 <55%		Ref.	—			Ref.	—							Ref.	—	
Ki-67 ≥55%		1,53	1.13, 2.08			1,4	0.79, 2.45							1,44	1.05, 1.98	
CRP cont	349	1	1.00, 1.01	**<0.001**	343	1	1.00, 1.00	0.357	349	1	1.00, 1.00	0.206				
CRP	349			**0.003**	343			0.83								
<10		Ref.	—			Ref.	—									
≥10		1,39	1.12, 1.74			1,03	0.79, 1.35									
CgA	424			0.259	343			0.895								
normal		Ref.	—			Ref.	—									
high		1,17	0.93, 1.47			0,97	0.73, 1.29									
not done		1,22	0.94, 1.59			0,92	0.66, 1.29									
LDH	425			**<0.001**	343			0.216	349			0.17				
normal		Ref.	—			Ref.	—			Ref.	—					
high		1,51	1.23, 1.86			1,27	0.97, 1.67			1,27	0.99, 1.64					
not done		1,23	0.86, 1.75			1,15	0.72, 1.84			1,11	0.72, 1.70					
ALP	425			**<0.001**	343			0.153	349			**0.017**	420			**0.004**
normal		Ref.	—			Ref.	—			Ref.	—			Ref.	—	
high		1,47	1.20, 1.79			1,28	1.00, 1.64			1,41	1.11, 1.79			1,43	1.15, 1.77	
not done		1	0.51, 1.96			1,25	0.48, 3.25			1,23	0.49, 3.10			1,04	0.53, 2.05	
Platelets	425			0.15	343			0.384	349			0.388				
normal		Ref.	—			Ref.	—			Ref.	—					
high		1,26	1.00, 1.59			1,03	0.77, 1.36			0,98	0.74, 1.29					
not done		1,35	0.50, 3.63			6,11	0.79, 47.5			6	0.79, 45.6					
Leucocytes	425			**<0.001**	343			0.334	349			0.209				
normal		Ref.	—			Ref.	—			Ref.	—					
high		1,53	1.24, 1.89			1,17	0.89, 1.55			1,24	0.94, 1.63					
not done		0,76	0.36, 1.61			0,5	0.10, 2.43			0,52	0.11, 2.47					

HR = Hazard Ratio, CI = Confidence Interval CUP= unknown primary PS= perfromance status. cont= continous, CgA=chromogranin A staining, LDH=lactate dehydrogenase, ALP=alkaline phosphatase

Table S7. Univariate and multivariate analyses for PFS in NET G3.

	Univariable				Full model				Final model			
**Characteristic**	**N**	**HR**	**95% CI**	**p-value**	**N**	**HR**	**95% CI**	**p-value**	**N**	**HR**	**95% CI**	**p-value**
Age	116	1,01	0.99, 1.03	0.274	92	1,02	0.99, 1.04	0.15	109	1,01	0.98, 1.03	0.572
Sex	116			**0.078**	92			0.353	109			0.318
Male		Ref.	—			Ref.	—			Ref.	—	
Female		1,41	0.96, 2.06			1,26	0.78, 2.03			1,24	0.82, 1.87	
Primary tumor	116			0.203	92			0.709				
pancreas		Ref.	—			Ref.	—					
SI		0,71	0.41, 1.23			0,73	0.34, 1.57					
other		1,17	0.78, 1.75			0,89	0.54, 1.48					
PS	113			**0.045**	92			**0.005**	109			0.057
0		Ref.	—			Ref.	—			Ref.	—	
1		1,36	0.91, 2.05			1,78	1.06, 3.00			1,59	0.99, 2.56	
2		2,08	1.17, 3.69			3,02	1.51, 6.07			1,89	1.02, 3.48	
Ki-67 cont	116	1,01	1.00, 1.02	**0.023**	92	1,01	0.99, 1.03	0.419	109	1	0.99, 1.01	0.667
Ki-67	116			0.355	92			0.642				
Ki-67 <55%		Ref.	—			Ref.	—					
Ki-67 ≥55%		1,3	0.76, 2.21			0,75	0.22, 2.52					
Chemo	116			**<0.001**	92			**<0.001**	109			**<0.001**
no plat/eto		Ref.	—			Ref.	—			Ref.	—	
plat/eto		3,27	2.15, 4.97			2,84	1.56, 5.18			3,53	2.14, 5.80	
CRP	94	1	1.00, 1.01	0.103	92	1	1.00, 1.01	0.549				
ALP	112			**0.095**	92			0.195	109			0.585
normal		Ref.	—			Ref.	—			Ref.	—	
high		1,53	1.04, 2.27			1,35	0.84, 2.18			1,2	0.78, 1.85	
not done		1,18	0.36, 3.83			0,45	0.10, 2.12			0,73	0.16, 3.22	

HR = Hazard Ratio, CI = Confidence Interval, SI= small intestinal, PS= performance status, cont= continous, plat/eto= platinum/etoposide
ALP=alkaline phosphatase

Table S8. Univariate and multivariate analyses for OS in NET G3.

	Univariable				Full model				Final model			
**Characteristic**	**N**	**HR**	**95% CI**	**p-value**	**N**	**HR**	**95% CI**	**p-value**	**N**	**HR**	**95% CI**	**p-value**
Age	116	1,03	1.01, 1.05	**0.003**	92	1,06	1.03, 1.09	**<0.001**	109	1,05	1.02, 1.08	**<0.001**
Sex	116			0.273	92			0.53	109			0.504
Male		Ref.	—			Ref.	—			Ref.	—	
Female		1,25	0.84, 1.86			0,86	0.54, 1.38			0,86	0.56, 1.33	
Primary tumor	116			0.132	92			0.967	109			0.2
pancreas		Ref.	—			Ref.	—			Ref.	—	
SI		0,61	0.33, 1.12			0,91	0.39, 2.15			0,65	0.33, 1.29	
other		1,11	0.73, 1.71			1,01	0.59, 1.75			1,19	0.73, 1.94	
PS	113			**<0.001**	92			**<0.001**	109			**0.007**
0		Ref.	—			Ref.	—			Ref.	—	
1		1,48	0.95, 2.30			1,72	0.98, 3.03			1,45	0.88, 2.39	
2		3,55	1.95, 6.47			4,71	2.14, 10.4			2,97	1.54, 5.72	
Ki-67 cont	116	1,01	1.00, 1.02	**0.008**	92	1	0.98, 1.03	0.851	109	1,01	0.99, 1.03	0.444
Ki-67	116			**0.079**	92			0.464	109			0.673
Ki-67 <55%		Ref.	—			Ref.	—			Ref.	—	
Ki-67 ≥55%		1,7	0.97, 2.95			1,7	0.41, 7.01			1,31	0.38, 4.56	
Chemo	116			**<0.001**	92			**0.045**	109			**0.007**
no plat/eto		Ref.	—			Ref.	—			Ref.	—	
plat/eto		2,03	1.36, 3.05			1,72	1.01, 2.93			1,83	1.18, 2.84	
CRP cont	94	1	1.00, 1.01	**0.058**	92	1	1.00, 1.01	0.301				
ALP	112			**<0.001**	92			**<0.001**	109			**<0.001**
normal		Ref.	—			Ref.	—			Ref.	—	
high		2,41	1.56, 3.72			2,74	1.54, 4.89			2,74	1.70, 4.43	
not done		0,98	0.30, 3.20			0,54	0.12, 2.55			0,59	0.13, 2.64	

HR = Hazard Ratio, CI = Confidence Interval, SI=small intestinal, PS= perfromance status. cont= continous, ALP=alkaline phosphatase, plat/eto= platinum/etoposide
